# Using LLM-generated tools to extract information about reporting statistical software in biomedical and health science research articles

**DOI:** 10.1186/s13104-026-07908-1

**Published:** 2026-06-27

**Authors:** Diego A. Forero, Pentti Nieminen

**Affiliations:** 1 School of Health and Sport Sciences, Fundación Universitaria del Área Andina, Bogotá, Colombia; 2 Medical Informatics and Statistics Research Group, University of Oulu, Oulu, Finland

**Keywords:** Biostatistics, Medicine, Health Sciences, Generative artificial intelligence, Statistical software, Study designs, Publications, Statistical reporting

## Abstract

**Objective:**

A major problem with reviewing the statistical methodology in published medical articles is that extracting the necessary details from large sample sets is time-consuming. This paper demonstrates how a novel automated procedure can extract information about statistical reporting from literature. To illustrate this, we searched the PubMed Central database for original research articles published in 2021 and 2023 to identify the statistical software packages used for data analysis. A key element in terms of transparency and reproducibility is the reporting of the software used for statistical analysis.

**Results:**

A freely available Shiny App was created with the help of generative artificial intelligence, and it was used to retrieve automatically information from randomly selected samples of articles indexed in PubMed Central. We analyzed a large sample of articles (*n* = 1740) to determine the reporting of statistical software for nine study designs. We found that, across different study types, proprietary software such as IBM SPSS Statistics still dominates. Despite multiple calls for greater use of open-source research software, these programs are not used as frequently. In addition, a surprising number of articles did not report the software used. Furthermore, this is the first application of the recent Vibe Coding concept to statistical research methods.

**Supplementary Information:**

The online version contains supplementary material available at 10.1186/s13104-026-07908-1.

## Introduction

Adequate use of basic and advanced biostatistical methods has been a cornerstone of health and biomedical sciences research, allowing the analysis of large datasets for the identification of efficacy of treatments, risk factors and diagnostic approaches, among others [1–3]. In this context, statistical software has been a fundamental engine as the analysis of those large datasets would not be possible without the implementation of the statistical techniques and significance tests in computer programs [2, 3].

The quality of scientific literature in the health and biomedical sciences depends on adequate reporting of methods and results [4, 5]. It is important to identify the statistical software package used in the data analysis because different statistical programs use different algorithms and default options to compute statistics [6]. Consequently, the findings may differ depending on the software package or algorithm used. Reporting the statistical software used in the methods section also helps readers to validate the authors’ findings [4]. Few previous studies, using manual extraction methods, have shown that the use of statistical methods and reporting practices varies between journals, even among medical subfields [7, 8].

Recently, there has been a great interest in exploring the potential of the application of generative Artificial Intelligence tools to research [9] in the health sciences, particularly regarding the use of Large Language Models (LLMs) [10].

This study aimed to assess the prevalence, using a novel LLM-generated tool, of different statistical software reported in clinical, biomedical, and health science research articles featuring a variety of study designs. Additionally, we aimed to provide recommendations for authors preparing manuscripts.

## Main text

### Methods

Initially, a pilot test of four commonly used LLMs was carried out, to evaluate their potential for extracting information about statistical software reported in articles indexed in PubMed Central [11]. Four free LLMs were tested: ChatGPT (OpenAI) [12], Gemini (Google) [13], DeepSeek (DeepSeek) [14] and Grok (xAI) [15].

#### Selection of articles

The PubMed Central [11] database was searched to identify indexed articles for nine types of study designs applied in clinical, biomedical and health sciences studies [16, 17]: Cell models, animal models, cross-sectional studies, case-control studies, cohort studies, randomized clinical trials, quasi-experimental studies, reliability and validation studies, and meta-analyses. The PubMed Central database was set up to comprehensively select articles whose full text is available (in many cases, the information about the software used is not available in the abstracts of the articles). As with previous articles [8, 18], specific years were selected and the search was carried out for studies published in 2021 and 2023 (Supplementary Table S2 presents the complete search strategies implemented). The target was to randomly select one hundred articles for each year and study design, which would result in a total of 1800 articles. Random numbers for study selection were generated using the *RAND* function in MS Excel 365 (Microsoft Corporation, Redmond, WA).

#### AI-powered development of an automated tool to extract information

Inspired by the very recent concept of *Vibe Coding* [19], several LLMs were tested for a prompt oriented to the development of a Shiny App [20] able to extract information about reported statistical software. After several rounds of revision of the AI-generated computer codes, and of the results of the resulting Shiny Apps, by a human expert, Gemini [13] and Claude [21] were the free LLMs selected for the automatic creation and refinement of the R code (the other two LLMs generated Shiny Apps with a lower performance). The free RStudio program (version 2025.05.0), running the open R language (version 4.4.3), was used to test the generated prototypes.

#### Measures

The final version of the AI-powered Shiny App was used to extract the statistical software reported in the selected article set. One of its main features is based on the *str_detect* function, from the *stringr* R package, and the use of lists of regular expressions with the common versions of statistical software. For quality control, in addition to the manual verification of the R code by a human expert, a manual verification of the screened data was done and, in cases of incorrect or incomplete data, it was manually confirmed by a human expert.

To carry out an exploratory analysis of some potential factors associated with the lack of reported software, we hypothesized that two main features of journals (ranking and/or turnaround times) might affect the reporting of software. Random subsamples of three main categories of articles were selected and extracted (a total of 226 articles): 1: Reporting IBM SPSS Statistics (hereinafter referred to as SPSS); 2: Reporting R, 3: Statistical software not reported. Information about turnaround times of the articles (number of days between submission of the manuscript and its acceptance by the journal) [22, 23] was extracted in PubMed Central for each article. In addition, to explore whether differences in the ranking, or visibility, of the journals might have an effect [8], data about quartiles of the Scopus ranking of the journals was extracted from the Scimago database (version 2024) [24].

#### Statistical analysis

Cross-tabulation was used to report the frequency and percentage distributions of the statistical software used in the evaluated articles by the study design. The Shapiro-Wilk test [25] was used to analyze whether the continuous turnaround times variable was normally distributed. As it was found not to have a normal distribution, the non-parametric Kruskal-Wallis test [26] was employed to statistically compare the value distributions of turnaround times between three article groups reporting SPSS, R or not reporting statistical software. Cross-tabulation was used to report differences in the frequency distributions of the categories of the journals in the Scimago database across the three journal groups. The chi-squared test [27] was used to evaluate the statistically significant differences in the distributions. A *p* value < 0.05 was defined as statistically significant. Box plots were used to visualize continuous data, while bar plots were used for categorical data. The open JASP statistical software (version 0.18.3.0) [28] was used for the statistical analyses.

### Results

A pilot exploration of four free LLMs, identified a large number of confabulations, or hallucinations, in their outputs (Table 1**)**. The LLMs provided erroneous answers, for example: they reported that narrative reviews used statistical software (it seems they were completely hallucinated answers as those programs were not mentioned in the full texts) or provided other programs not used in the selected articles.

**Table 1:** A brief example of the multiple confabulations found when free versions of four LLMs were asked to identify the statistical software used in articles indexed in PubMed Central (5 PMCIDs were provided)

Article	Type of Article	Actual Software	ChatGPT	Gemini	DeepSeek	Grok
PMC8450723	Narrative review	None	R 3.3.0; SAS 9.3	SPSS 25.0	R	R; SPSS
PMC11262073	Narrative review	None	SAS 9.4; R 4.1.2	SPSS 29.0	None	None
PMC8725884	Meta-analysis	OpenMeta[Analyst]	SPSS; R; Python; Stata; SAS	SPSS 25.0	SPSS	SPSS
PMC9035035	Randomized clinical trial	SAS 9.4	SPSS; SAS; Stata	SPSS 26.0	R	None
PMC8740153	Animal model	GraphPad Prism 8.0	SPSS; SAS; Stata	GraphPad Prism 8.0	Stata	None

#### An LLM-generated tool applied to biostatistics research

**Fig. 1:**
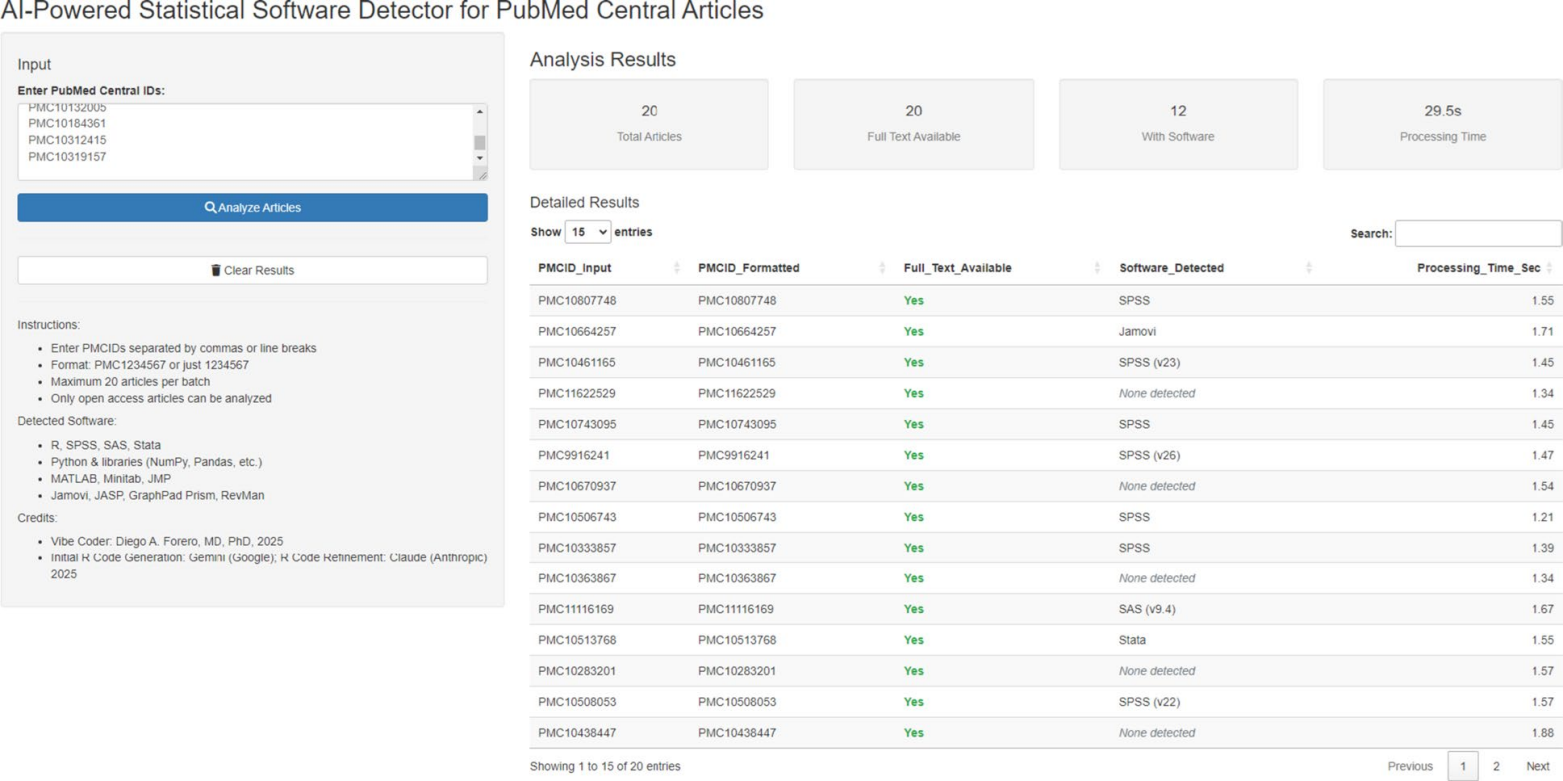
A screenshot of the AI-Powered Shiny App, developed with the concept of Vibe Coding and created to extract information about statistical software reported in full text articles indexed in PubMed

With the help of two LLMs, a new Shiny App was created to extract the statistical software reported in the selected articles from PubMed Central. The Shiny App takes advantage of the availability of several powerful and open R packages, created by human experts, such as the *shiny*, *tidyPMC* and *xml2* packages **(**Table 2**).** After several stages of revision and refinement by a human expert, the new Shiny App (Fig. 1) is freely available at shinyapps.io (daforerog.shinyapps.io/StatSoftPMC) and the respective final R code is available at GitHub.com (github.com/daforerog/StatSoftPMC, under a MIT License).

**Table 2:** An overview of the R packages incorporated into the Shiny App

*R* Package	Main use	Citation
**shiny**	*Makes it easy to build interactive web applications with R.*	Chang W, Cheng J, Allaire J, Sievert C, Schloerke B, Xie Y, Allen J, McPherson J, Dipert A, Borges B (2025). shiny: Web Application Framework for R. R package version 1.10.0.9001
**shinyjs**	*Performs common useful JavaScript operations in Shiny apps*	Attali D (2025). shinyjs: Easily Improve the User Experience of Your Shiny Apps in Seconds. R package version 2.1.0.9011,
**tidypmc**	*Parses XML documents from the Open Access subset of Europe PubMed Central*	Stubben C (2025). tidypmc: Parse Full Text XML Documents from PubMed Central. R package version 1.8,
**xml2**	*Allows the parsing of XML documents*	Wickham H, Hester J, Ooms J (2025). xml2: Parse XML. R package version 1.3.8,
**dplyr**	*Provides a grammar of data manipulation*	Wickham H, François R, Henry L, Müller K, Vaughan D (2023). dplyr: A Grammar of Data Manipulation. R package version 1.1.4,
**DT**	*Allows creating interactive HTML tables from R data*	Xie Y, Cheng J, Tan X (2025). DT: A Wrapper of the JavaScript Library ‘DataTables’. R package version 0.33.3
**stringr**	*Makes string manipulation simpler*	Wickham H (2023). stringr: Simple, Consistent Wrappers for Common String Operations. R package version 1.5.1
**purrr**	*Potentiates functional programming in R*	Wickham H, Henry L (2025). purrr: Functional Programming Tools. R package version 1.0.4

#### Statistical software reported in the evaluated set of articles indexed in PubMed Central

A total of 1740 biomedical and health sciences articles, indexed in PubMed Central, were assessed 200 articles were retrieved for each study design (9 study designs), except for cell models, where 140 articles were found for the two years selected. Our analysis revealed that, overall, the commercial SPSS software was the most frequently used (37.1%), followed by the open-source R packages (14.6%) and the proprietary Stata software (12.5%) (Table 3). Other programs were used less frequently. Of special interest, in terms of quality of reporting, almost 9% percent of the articles did not report statistical software (Table 3).

**Table 3:** A general overview of the statistical software reported for a large sample of articles (n: 1740) in the health and biomedical sciences and indexed in PubMed Central

Software	Type	Percent reported	Developer	Key article
SPSS	Commercial	37.1	IBM	Ayyanar K. SSRN, 2016
R	Open software	14.6	R Foundation	Wilson J. WIREs Comput Stat, 2012
Stata	Commercial	12.5	StataCorp	Gutierrez RG. WIREs Comput Stat, 2010
None	–	8.9	–	–
GraphPad Prism	Commercial	8.8	Dotmatics	Swift ML. J Chem Inf Comput Sci, 1997
SAS	Commercial	7.6	SAS Institute	Rodriguez RN. WIREs Comput Stat. 2011
RevMan	Commercial	4.2	Cochrane Collaboration	Tantry TP. Korean J Anesthesiol. 2021
Statistica	Commercial	0.9	TIBCO Software	Hilbe JM. Am Stat. 2007
MATLAB	Commercial	0.6	MathWorks	Sobie EA. Sci Signal. 2011
Mplus	Commercial	0.6	Muthén & Muthén	Vandenberg RJ. Organ Res Methods. 2006
CMA	Commercial	0.5	Biostat	Brüggemann P. J Mark Anal. 2022
Jamovi	Open software	0.5	jamovi project	Şahin M. Int J Assess Tool Educ. 2019
JMP	Commercial	0.5	SAS Institute	Jones B. WIREs Comput Stat. 2011
JASP	Open software	0.3	JASP project	Love J, et al. J. Stat. Softw. 2019
MedCalc	Commercial	0.3	MedCalc Software	Schoonjans FR. Comput Methods Programs Biomed. 1995

**Fig. 2:**
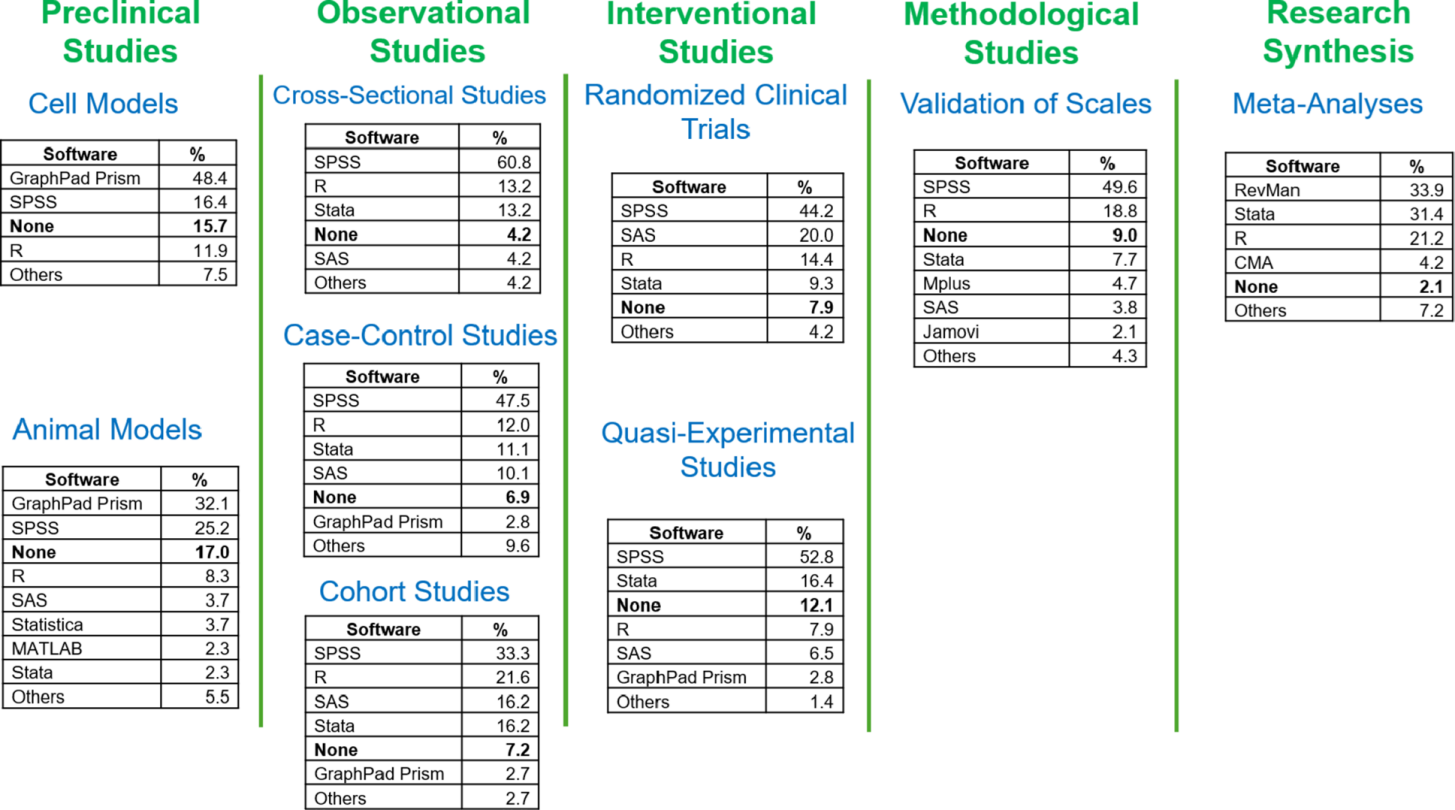
An overview of the distribution of statistical software reported for samples of nine different types of study design in the health and biomedical sciences

Taking into consideration the differences in standards between fields and research topics, an analysis stratified by study design found major variations (Fig. 2; please see Table S1 for further details). For preclinical research, such as cell models and animal models, the proprietary GraphPad Prism software was the most frequently reported (48.4%). In the context of research synthesis, the proprietary RevMan was the most commonly reported (33.9%), followed by Stata (31.4%). In observational studies (such as cross-sectional, case-control and cohort studies), SPSS (60.8, 47.5 and 33.3%, respectively) and R (13.2, 12.0 and 21.6%, respectively) were the most used programs. The lack of reporting statistical software was higher for animal and cell models and for quasi-experimental studies (17.0, 15.7 and 12.1%, respectively) (Fig. 2).

#### Exploratory analysis of factors associated with the lack of reporting of statistical software

**Fig. 3:**
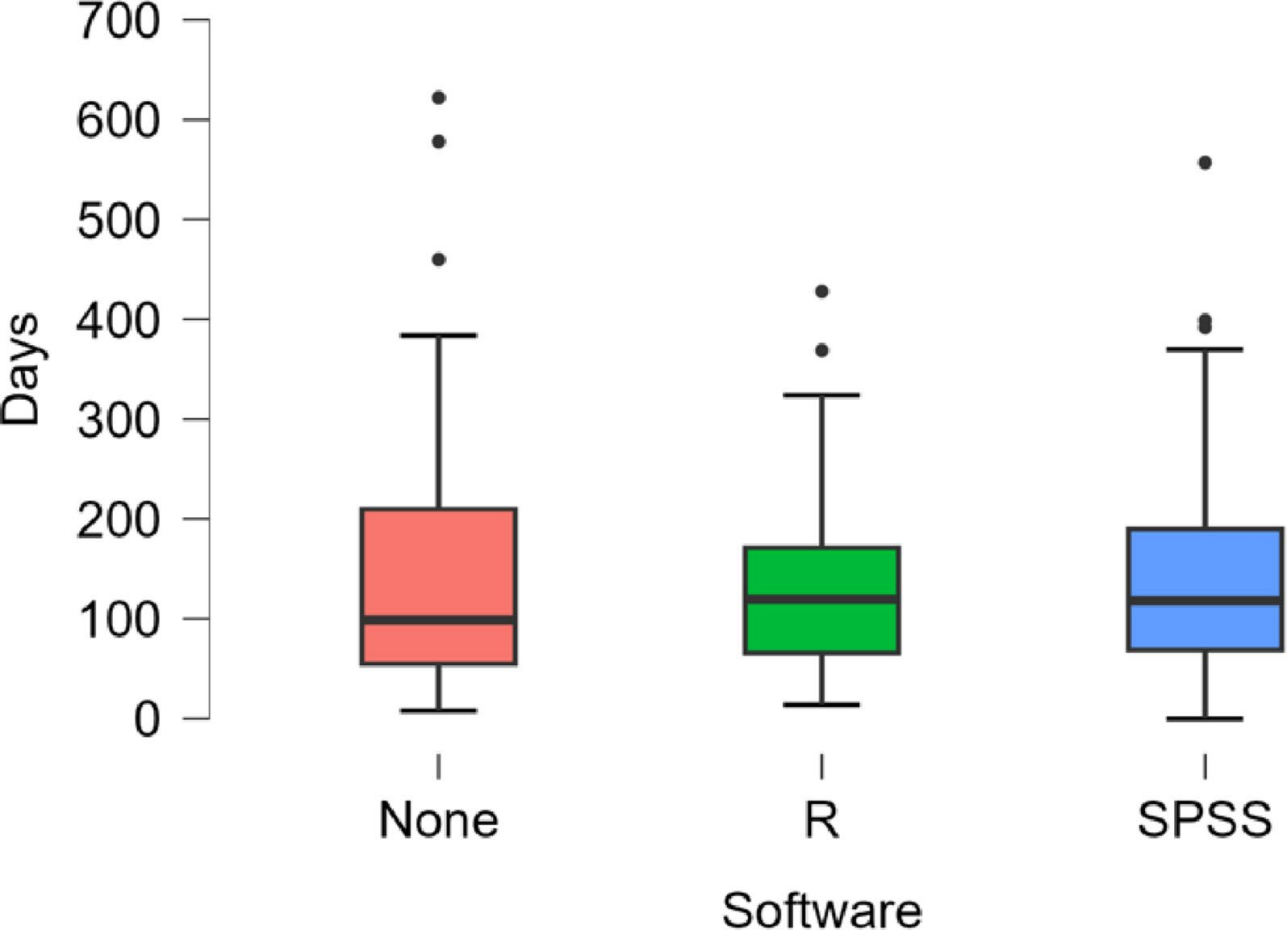
Box plot for the distribution of turnaround times (Y axis shows the number of days from submission to acceptance of the manuscript) for random samples of articles that did not report statistical software or that reported R or SPSS software. The boxes show the first and third quartiles, with the thick horizontal lines highlighting the medians; the whiskers show the minimum and maximums. The result from a Kruskal-Wallis test, for a comparison between the 3 groups, was non-significant (*p* value: 0.741)

**Fig. 4:**
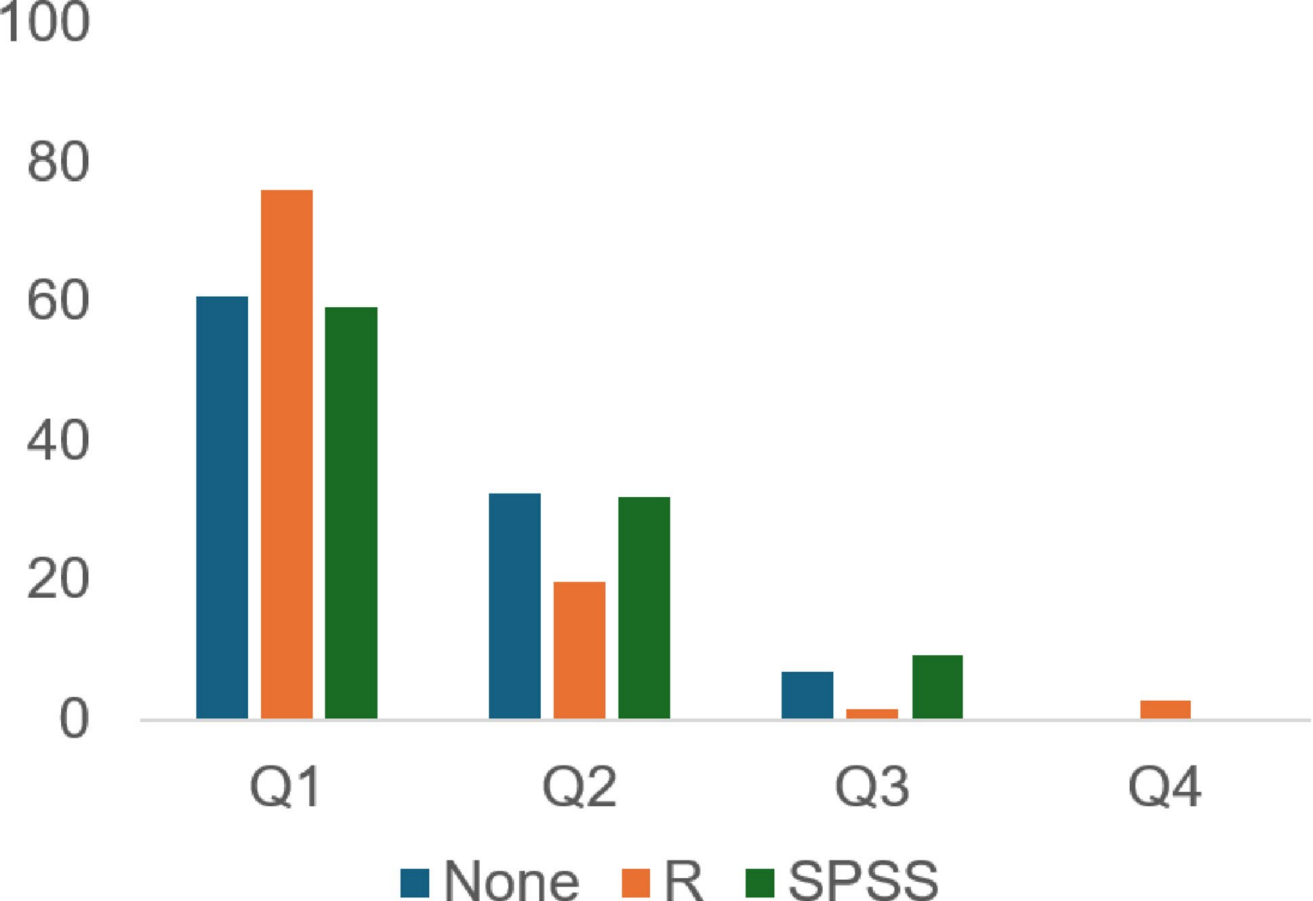
Bar plot for the distribution of quartiles for the journals (extracted from Scimago 2024) of random samples of articles (a total of 226 articles) that did not report statistical software or that reported R or SPSS software. The Y axis shows the percentage of journals in each one of the four Scimago quartiles. The result from a chi-squared test, for a comparison between the three groups, was non-significant (*p* value: 0.058)

In an exploratory analysis, we analyzed two main factors potentially associated with the absence of statistical software reported in articles. The turnaround times were not significantly different in the sample of articles without statistical software in comparison with the samples of articles reporting SPSS or R (Fig. 3) and the distribution of quartiles of the journals, according to the international Scopus ranking, was also not significantly different between the three groups (Fig. 4).

Finally, in order to provide a valuable resource for scientists around the world, we consolidated the recommended citations for the current versions of the most frequently used statistical programs (Table 4).

**Table 4:** Examples of recommended citation styles for the recent versions of the most commonly used proprietary and open statistical software

Software	Recommended citation
SPSS	IBM Corp. Released 2023. IBM SPSS Statistics for Windows, Version 29.0.2.0 Armonk, NY: IBM Corp
Stata	StataCorp. 2025. Stata Statistical Software: Release 19. College Station, TX: StataCorp LLC.
GraphPad Prism	GraphPad Prism version 10.0.0 for Windows, GraphPad Software, Boston, Massachusetts USA
SAS	SAS^®^ software, Version 9.4 for Windows. Copyright © 2024, SAS Institute Inc
R	R version 4.4.1 (R Core Team, 2024), RStudio (Rstudio Team, 2025), and the tidyverse package (Wickham, 2019).
Jamovi	The jamovi project (2025). jamovi (Version 2.6) [Computer Software]. Retrieved from https://www.jamovi.org
JASP	JASP Team (2024). JASP (Version 0.19.3) [Computer software].

### Discussion

We were able to obtain data from a large number of published studies for further analysis with the help of the described LLM-generated Shiny App tool. Our findings highlight a differential use, depending on the study design, of statistical programs and identify a relatively high number of articles that did not report statistical software. Our preliminary analyses found that the lack of reporting statistical software was not associated with turnaround times of their articles or with quartiles of an international ranking of the journals [8].

Our current results complement and extend the findings from some previous articles, which used manual methods for data extraction [7, 8, 18, 29]. Our wide-ranging study shows that differences in reporting practices and analytical statistical tools still exist between various fields related to medicine and other health sciences. Statistical demands are different between basic and clinical research [30, 31]. Animal studies and basic biomedical research typically involve experimental designs with a high degree of invasiveness, less intraindividual variation, and small sample sizes [31, 32].

The statistical software used in the analysis should be reported in every article containing statistical analyses [4]. Several study designs from the health sciences research commonly use proprietary software (such as SPSS, Stata and SAS) [8], which are used, almost daily, by biostatisticians and the licenses for these programs are paid by the institutions). On the other hand, for a researcher who needs statistical software occasionally, they may not be suitable and there is the opportunity for user-friendly and freely available software [33].

In recent years, problems in the reproducibility of biomedical research have received considerable attention [34]. These issues have been linked to the poor presentation of statistical findings and the inadequate reporting of data analysis techniques by authors [4, 35]. The lack of reporting of statistical software, particularly for designs such as animal models, randomized clinical trials and quasi-experimental studies, is of particular concern, due to the clinical implications of their results in humans or the effects on potential research waste [36] and ethical considerations in animal models. Peer reviewers and editors should have special attention to these aspects in the methods of submitted manuscripts [4, 7]. However, our results suggest that progress has been made in how software tools are reported. Previous studies have found significantly higher proportions of inadequate reporting of statistical software in medical articles [37–40].

Our results, showing an overall low use of open-source software, call for promoting a higher use of these available programs, which have several advantages, such as the inclusion of powerful modules for advanced analyses (machine learning, for example), among others [33]. In addition, there is a need for an improvement for the complete and adequate reporting of R packages in articles, in order to provide the full information of all the R packages used in the studies [41] (an important number of articles only reported R and its version or RStudio).

This is one of the first studies in the health sciences taking advantage of the recent concept of Vibe Coding [19, 42], allowing the automatic creation of a novel computerized tool designed to be used in research on biostatistics. One of the very few existing original articles about Vibe Coding recently highlighted the importance of the manual checking of results arising from LLM-generated tools and the need for an adequate automatic organization and annotation of code [43], as we did in the current study.

Future development of statistical software should have into account several considerations, such as the importance of being user-friendly [44], the importance of including recent methods [45–47] and an adherence to the recent FAIR (Findable, Accessible, Interoperable and Reusable) principles for research software, which means that programs are easy for both humans and machines to find and reusable, among others [48].

### Limitations

A potential limitation of this work was the focus on articles indexed in PubMed Central. It was used to have access to the full texts of articles, which is needed for identification of reported statistical software. Another potential limitation was the selection of articles published in two years, as it was needed to guarantee a large sample of original papers with more homogenous features.

## Supplementary Information

Supplementary Material 1.

Supplementary Material 2.

## Data Availability

All data generated or analyzed during this study are included in this published article and its supplementary information files.
