# Supplementary material for: Using LLM-generated tools to extract information about reporting statistical software in biomedical and health science research articles
Source: BMC Res Notes. 2026 Jun 27;19:286. doi: 10.1186/s13104-026-07908-1 (PMC13352756; doi:10.1186/s13104-026-07908-1)
Supplement: Supplementary file 1 — Supplementary Material 1. [file 13104_2026_7908_MOESM1_ESM.docx]

| **Statistical Software** | **Cell Models n (%)** | **Animal Models n (%)** | **Cross-Sectional Studies n (%)** | **Case-Control Studies n (%)** | **Cohort Studies n (%)** | **Randomized Clinical Trials n (%)** | **Quasi-Experimental Studies n (%)** | **Validation of Scales n (%)** | **Meta-Analyses n (%)** |
| --- | --- | --- | --- | --- | --- | --- | --- | --- | --- |
| **SPSS** | 26 (16.4) | 55 (25.2) | 129 (60.8) | 103 (47.5) | 74 (33.3) | 95 (44.2) | 113 (52.8) | 116 (49.6) |  |
| **GraphPad Prism** | 77 (48.4) | 70 (32.1) |  | 6 (2.8) | 6 (2.7) |  | 6 (2.8) |  |  |
| **R** | 19 (11.9) | 18 (8.3) | 28 (13.2) | 26 (12) | 48 (21.6) | 31 (14.4) | 17 (7.9) | 44 (18.8) | 50 (21.2) |
| **Stata** |  | 5 (2.3) | 28 (13.2) | 24 (11.1) | 36 (16.2) | 20 (9.3) | 35 (16.4) | 18 (7.7) | 74 (31.4) |
| **None** | 25 (15.7) | 37 (17) | 9 (4.2) | 15 (6.9) | 16 (7.2) | 17 (7.9) | 26 (12.1) | 21 (9) | 5 (2.1) |
| **SAS** | 3 (1.9) | 8 (3.7) | 9 (4.2) | 22 (10.1) | 36 (16.2) | 43 (20) | 14 (6.5) | 9 (3.8) |  |
| **Statistica** |  | 8 (3.7) |  | 4 (1.8) |  |  |  |  |  |
| **MATLAB** | 2 (1.3) | 5 (2.3) |  |  |  |  |  |  |  |
| **RevMan** |  |  |  |  |  |  |  |  | 80 (33.9) |
| **CMA** |  |  |  |  |  |  |  |  | 10 (4.2) |
| **Mplus** |  |  |  |  |  |  |  | 11 (4.7) |  |
| **Jamovi** |  |  |  |  |  |  |  | 5 (2.1) |  |
| **JASP** |  |  |  |  |  |  |  | 3 (1.3) |  |
| **Others** | 7 (4.5) | 12 (5.5) | 9 (4.2) | 15 (6.9) | 6 (2.8) | 8 (3.7) | 3 (1.4) | 6 (2.6) | 17 (7.2) |

**Table S1.** Detailed information on statistical software reported, according to study design.

There were 200 articles analyzed per study design, except for cell models, where there were 140 articles for the two years selected. If more than one software program was reported in an article, all reported programs were included in our data.

| **Study Design** | **Search terms** |
| --- | --- |
| **Cell models** | (cell model[Title]) AND (("2021/01/01"[Date - Publication] : "2021/12/31"[Date - Publication]))  (cell model[Title]) AND (("2023/01/01"[Date - Publication] : "2023/12/31"[Date - Publication])) |
| **Animal models** | (animal model[Title]) AND (("2021/01/01"[Date - Publication] : "2021/12/31"[Date - Publication]))  (animal model[Title]) AND (("2023/01/01"[Date - Publication] : "2023/12/31"[Date - Publication])) |
| **Case-control studies** | (case-control study[Title]) AND (("2021/01/01"[Date - Publication] : "2021/12/31"[Date - Publication]))  (case-control study[Title]) AND (("2023/01/01"[Date - Publication] : "2023/12/31"[Date - Publication])) |
| **Cross-sectional studies** | (cross-sectional study[Title]) AND (("2021/01/01"[Date - Publication] : "2021/12/31"[Date - Publication]))  (cross-sectional study[Title]) AND (("2023/01/01"[Date - Publication] : "2023/12/31"[Date - Publication])) |
| **Cohort studies** | (cohort study[Title]) AND (("2021/01/01"[Date - Publication] : "2021/12/31"[Date - Publication]))  (cohort study[Title]) AND (("2023/01/01"[Date - Publication] : "2023/12/31"[Date - Publication])) |
| **Randomized clinical trials** | (randomized clinical trial[Title]) AND (("2021/01/01"[Date - Publication] : "2021/12/31"[Date - Publication]))  (randomized clinical trial[Title]) AND (("2023/01/01"[Date - Publication] : "2023/12/31"[Date - Publication])) |
| **Quasiexperimental studies** | (quasiexperimental study[Title]) AND (("2021/01/01"[Date - Publication] : "2021/12/31"[Date - Publication]))  (quasiexperimental study[Title]) AND (("2023/01/01"[Date - Publication] : "2023/12/31"[Date - Publication])) |
| **Scale validation studies** | ((scale[Title]) AND (validation[Title])) AND (("2021/01/01"[Date - Publication] : "2021/12/31"[Date - Publication]))  ((scale[Title]) AND (validation[Title])) AND (("2023/01/01"[Date - Publication] : "2023/12/31"[Date - Publication])) |
| **Meta-analyses** | (meta-analysis[Title]) AND (("2021/01/01"[Date - Publication] : "2021/12/31"[Date - Publication]))  (meta-analysis[Title]) AND (("2023/01/01"[Date - Publication] : "2023/12/31"[Date - Publication])) |

**Table S2. Description of the search terms implemented in PubMed Central for the nine study designs.**
